# Supramolecular Crystal Networks Constructed from Cucurbit[8]uril with Two Naphthyl Groups

**DOI:** 10.3390/molecules28010063

**Published:** 2022-12-21

**Authors:** Zhong-Zheng Gao, Lei Shen, Yu-Lu Hu, Ji-Fu Sun, Gang Wei, Hui Zhao

**Affiliations:** 1 College of Chemical and Biological Engineering, Shandong University of Science and Technology, 579 Qianwangang Road, Qingdao 266590, China; 2 Commonwealth Scientific and Industrial Research Organisation (CSIRO), Mineral Resources, P.O. Box 218, Lindfield, NSW 2070, Australia

**Keywords:** cucurbit[8]uril, naphthyl group, host–guest chemistry, self-assembly, supramolecular network

## Abstract

Naphthyl groups are widely used as building blocks for the self-assembly of supramolecular crystal networks. Host–guest complexation of cucurbit[8]uril (Q[8]) with two guests **NapA** and **Nap1** in both aqueous solution and solid state has been fully investigated. Experimental data indicated that double guests resided within the cavity of Q[8], generating highly stable homoternary complexes **NapA**_2_@Q[8] and **Nap1**_2_@Q[8]. Meanwhile, the strong hydrogen-bonding and π···π interaction play critical roles in the formation of 1D supramolecular chain, as well as 2D and 3D networks in solid state.

## 1. Introduction

Supramolecular self-assembly is a spontaneous process in which basic structural units form ordered structures driven by non-covalent interactions (including electrostatic interactions, van der Waals forces, hydrogen bond, π–π stacking, hydrophobic effects, etc.) [1,2,3,4,5,6]. Based on self-assembly, various supramolecular network structures have been widely developed. Reversible non-covalent bonds endow supramolecular networks with traditional polymer properties, as well as dynamic properties, which can achieve high response to stimuli and self-healing functions [7,8,9,10]. These supramolecular network systems have been constructed to synthesize different functional materials and widely used in drug carriers, nano containers, molecular devices, sensors, adsorption and separation materials, catalysts, and environmental pollutant treatment [11,12,13,14,15]. Although the supramolecular polymerization process has been deeply understood, it is still a huge challenge to design controllable supramolecular polymerization systems and provide supramolecular polymers with controllable structures [16].

The macrocyclic host cucurbit[n]urils (Q[n]s, CB[n]s, n = number of glycoluril units) are spherical macrocyclic compounds adopting a stable rigid structure and composed of *n* glycoluril units bridged by *2n* methylene groups with a hydrophobic cavity [17,18,19,20]. As a kind of rigid macrocyclic host, cucurbit[n]urils have three main structural characteristics, namely, the neutral cavity, the negative electrostatic potential carbonyl portals and the positive electrostatic potential of the outer surface [21,22]. Based on the above three characteristics, the Q[n]s related host-guest chemistry, coordination chemistry and outer surface interaction chemistry have been widely used [23,24,25]. The cavity of the Q[n]s can selectively bind a part of or whole guest molecules to form host–guest complexes [26,27,28,29]. According to the Q[n]’s cavity size, Q[8] exhibits unique molecular recognition characteristics enabling dimerization of specific guest molecules inside the cavity of Q[8] in a controlled manner. The larger cavity of Q[8] can bind two aromatic group, such as phenyl, naphthyl, indole, quinoline, and larger aromatic rings, thus allowing for the formation of homoternary inclusion complexes [30,31,32]. Therefore, Q[8] has been vigorously studied as basic units for the construction of supramolecular networks [33,34,35]. For example, Zhang’s team constructed a variety of supramolecular hyperbranched networks by self-assembly of dendrimers and Q[8] [36,37]. Liu’s team constructed a variety of two-dimensional supramolecular network structures based on triphenylamine and Q[8] [38,39], which have good effects in many fields such as cell imaging, near-infrared lysosome targeted imaging. Li’s team prepared a tetrahedral molecule which was used to co-assemble with Q[8] to afford a new water soluble 3D diamondoid system [40]. Moreover, the hexa-armed [Ru(bpy)_3_]^2+^-based derivatives and Q[8] were used to afford another 3D cubelike system, which could be used as heterogeneous catalyst for hydrogen production and organic reaction [41]. In previous work, we used the antiparallel encapsulation of styrene pyridine dimer in Q[8] to construct a variety of supramolecular polymer systems [42,43]. For example, we generated an irreversible covalent component for the construction of the first highly watersoluble 3D supramolecular-covalent organic framework, which was used to highly promote the electron transfer of protons to H_2_ when loaded on POM catalysts and Ru^2+^-complex photosensitizers [42]. Having said that, we believe that Q[n]s are ideal as basic building blocks for the construction of Q[n]-based networks.

Herein, we introduced two guests containing naphthyl groups, 4-(4-carboxyphenyl)-1-(naphthalen-2-ylmethyl)pyridin-1-ium bromide (**NapA**) and 1-(naphthalen-2-ylmethyl)-[4,4′-bipyridin]-1-ium bromide (**Nap1**) for the formation of supramolecular networks. The Q[8]-induced supramolecular networks were investigated by ^1^H nuclear magnetic resonance (^1^H NMR) spectrum, UV-vis absorption spectrum, isothermal titration calorimetry (ITC), dynamic light scattering (DLS), and single-crystal X-ray crystallography. Experimental data indicated that double guests resided within the cavity of Q[8] in both aqueous solution and solid state, generating highly stable homoternary complexes **NapA**_2_@Q[8] and **Nap1**_2_@Q[8]. The strong hydrogen-bonding and π···π interaction played critical roles in the formation of supramolecular networks.

## 2. Results

### 2.1. Molecular Binding Behavior and Thermodynamic between Cucurbit[8]uril and **NapA** or **Nap1**

**Scheme 1:**
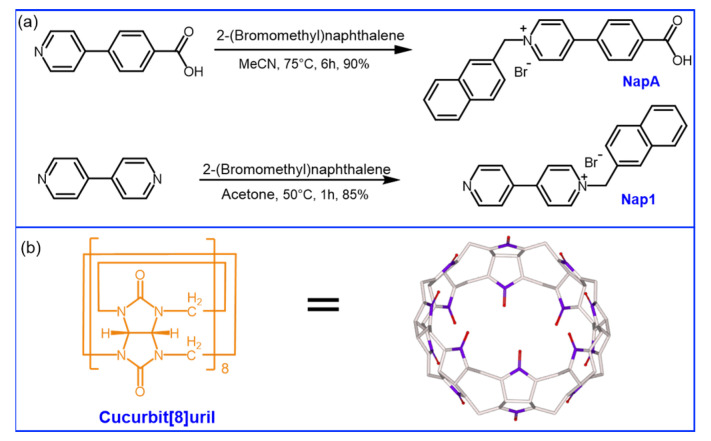
(**a**) The synthesis of **NapA** and **Nap1** and (**b**) the structure of host Q[8].

It has been established that Q[8] encapsulation for the dimers of naphthyl unit in water can remarkably promote the π–π interaction. Thus, two guests containing naphthyl unit (**NapA** and **Nap1**) were designed and synthesized (Scheme 1). Two guests, **NapA** and **Nap1,** were prepared in a one-step sequence. Compound **NapA** was prepared from the reaction of 4-(4-pyridyl)benzoic acid and 2-bromomethyl naphthalene in MeCN in 90% yield and characterized using ^1^H NMR, ^13^C NMR, ^1^H-^1^H COSY spectra and high-resolution mass spectra (HR-MS). 4,4′-bipyridine and 2-bromomethyl naphthalene were used to synthesize **Nap1** in acetone in 85% yield. We then studied their coassembly with Q[8] in water for the formation of two new homoternary supramolecular complexes.

The naphthyl units of two guests can combine to form supramolecular dimers under the action of Q[8]-driven self-assembly. Two examples of homoternary supramolecular complexes were constructed under the coordination of supramolecular interactions such as Q[n]s’ outer surface interaction, hydrogen bond interaction, strong π–π interaction, host-guest interaction, etc. Simply mixing Q[8] and **Nap1** or **NapA** in aqueous solution resulted in the formation of the supramolecular networks depending on the corresponding host–guest interaction between the two species of molecules, which was further characterized by ^1^H NMR spectroscopy, X-ray single crystal diffraction, and dynamic light scattering analysis (DLS).

**Figure 1:**
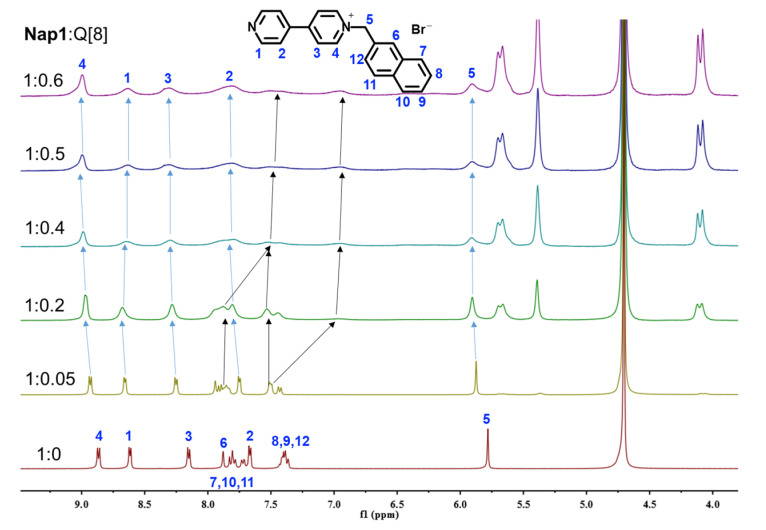
^1^H NMR spectrum (400 MHz) of the mixtures of **Nap1** (1.0 mM) and Q[8] (0 to 0.6 equiv) in D_2_O at 25 °C.

The ^1^H NMR spectroscopy measurements indicated that both **Nap1** and **NapA** form host-guest inclusion complexes with Q[8] host. In the presence of small amount of the Q[8] hosts (Figure 1), the signals of both free and complexed guests were simultaneously observed and were very broad, indicating slow exchange of free and complexed guests on the NMR time scale. On the one hand, the protons of bipyridine (H_1_-H_4_) and one of the CH_2_ protons (H_5_) of **Nap1** moved downfield slightly, which indicated that they were located outside the cavity. On the other hand, at a 2:1 ratio of **Nap1** to Q[8], the naphthyl peaks (H_6_-H_12_) were completely shifted upfield. These observations suggested that the naphthyl moiety of the Nap1 guest was encapsulated into the cavity of the Q[8] host.

The corresponding ^1^H NMR titration spectra of the Q[8] with **NapA** were showed in Figure S1. Due to the low solubility of **NapA**, the NMR signals became wide peak after adding Q[8]. In the presence of 0.5 equiv of Q[8], the signals corresponding to the naphthyl (H_6_-H_12_) protons of the NapA shifted upfield, while those corresponding to the pyridinyl (H_3_ and H_4_), phenyl (H_1_ and H_2_) protons did not move significantly. This clearly indicated that the naphthyl protons of the guest NapA were buried inside the hydrophobic cavity of Q[8], while the 4-(4-pyridyl)benzoic acid moiety resided outside of the Q[8] portals, forming homoternary supramolecular complexes with 1:2 host-guest binding ratio **NapA**_2_@Q[8]. By comparing the NMR titration data of the two self-assemblies, we found that the naphthyl group of the guest molecule was encapsulated in the hydrophobic cavity of Q[8].

**Figure 2:**
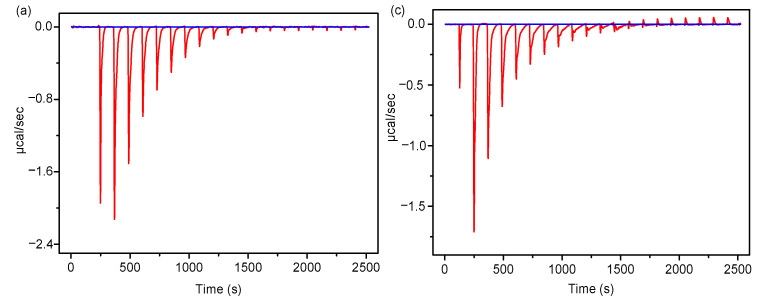

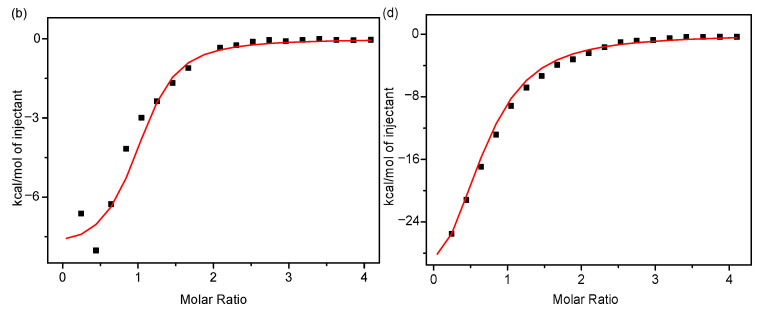
(**a**,**b**) **Nap1** (1.0 mM) and (**c**,**d**) **NapA** (1.0 mM) titration of the Q[8] (0.1 mM) isothermal titration heat curve and nonlinear fitting result of molar ratios at 25 °C.

Isothermal titration calorimetry (ITC) was also employed to afford the thermodynamic parameters of Q[8] with both **Nap1** and **NapA**. ITC experimental data further confirmed that the binding stoichiometry of Q[8] to both **Nap1** and **NapA** is 1:2 (Figure 2). From the Δ*H* and *T*Δ*S* values in Table S1, it was clear that the formation of both homoternary complexes were enthalpically driven. The observed negative enthalpy change (Δ*H* = −36.17 ± 2.59 kJ·mol^−1^ for **NapA**_2_@Q[8]; Δ*H* = −22.71 ± 1.37 kJ·mol−1 for **Nap1**_2_@Q[8]) were probably due to the cooperativity of above mentioned four kinds of weak interactions. On the basis of the corresponding experimental results, we also obtained the association constants of *K*_a_ = (2.14 ± 0.62) × 10^10^ M^−2^ and (1.48 ± 0.45) × 10^10^ M^−2^ for Q[8] with **NapA** and **Nap1**, respectively, which was much larger than that of Q[8] with tripeptides reported by Urbach [44] and closer to Q[8] with tripeptides reported by us [30]. Such a high binding constant suggested the relatively strong host–guest interaction between Q[8] and **NapA** or **Nap1**, indicating the construction of stable homoternary complexes **NapA**_2_@Q[8] and **Nap1**_2_@Q[8] in aqueous solution.

**Figure 3:**
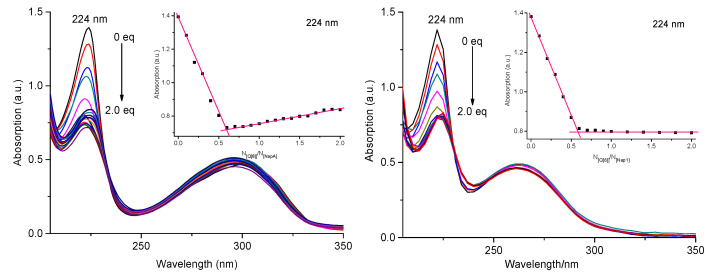
UV-vis spectra of **NapA** and **Nap1** (10 μM) with the addition of Q[8] (0–2.0 equiv.) in water at 25 °C (Inset: Absorbance at 224 nm vs. [Q[8]]/[**NapA**], and [Q[8]]/[**Nap1**]).

To better understand the host-guest interaction between Q[8] and both **Nap1** and **NapA** in aqueous solution, we also carried out UV-vis titration experiments. According to the UV-vis absorption spectroscopic results, as shown in Figure 3, the compound **Nap1** and **NapA** displayed an absorption band at 224 nm belonging to naphthyl unit, which decreased markedly in its intensity upon addition of Q[8], due to the strong interaction between Q[8] and naphthyl moiety of guest molecules **Nap1** and **NapA**. When 0.5 equivalent Q[8] was added, the UV−vis absorption spectra intensity did not change significantly. Their Job plots (based on the continuous variation method) clearly showed that UV−vis spectra data of both **Nap1** and **NapA** fitted well to 1:2 stoichiometry of the host-guest inclusion complexes (Figure 3, inset). The binding stoichiometry was also determined by the Job plot analysis at a fixed total concentration of host and guest molecules. The absorption intensity changes (Δ*A*) were plotted against the molar fraction of **Nap1** and **NapA** to give a peak at a molar fraction of 0.66, indicating a 1:2 stoichiometry for the Q[8]:**Nap1** or Q[8]:**NapA** inclusion complex (Figures S2 and S3), which was consistent with the NMR titration results.

**Figure 4:**
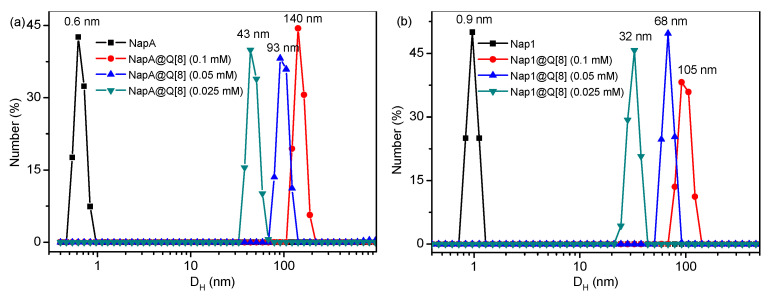
DLS profile of (**a**) **NapA**_2_@Q[8] and (**b**) **Nap1_2_**@Q[8] in water at 25 °C. The concentration represents that of **NapA** or **Nap1** of homoternary complexes.

DLS experiments in dilute solutions can be used to monitor the formation of supramolecular networks. DLS results revealed that **NapA**, **Nap1** and Q[8] formed nanoscaled assemblies in water. As can be found, the hydrodynamic diameter (D_H_) of **NapA** monomer (1.0 mM) was determined to be 0.6 nm. In Figure 4a, mixing Q[8] and **NapA** (1:2, [**NapA**] = 0.1 mM) observed one hydrodynamic diameter distribution centered at 140 nm. It showed that aggregates of this size were formed in the aqueous solution. The D_H_ value decreased with the dilution of the solution. However, even at [**NapA**] = 25 μM, D_H_ was still as high as 43 nm. These observations supported that **NapA** and Q[8] coassembled into the nanoscaled supramolecular networks in water. At the same concentration, the hydrated particle size of assembly **NapA**_2_@Q[8] is obviously higher than that of assembly **Nap1**_2_@Q[8], which indicates that the polymerization ability of assembly **NapA**_2_@Q[8] is higher than that of assembly **Nap1_2_**@Q[8] in the aqueous phase. DLS experiment showed that the self-assembly had a certain stability in the solution even at low concentration. The result was consistent with the results of crystal structure description. The DLS experiment of **Nap1**_2_@Q[8] (Figure 4b) was similar to that of **NapA**_2_@Q[8].

### 2.2. Single-Crystal X-ray Crystallography

**Figure 5:**
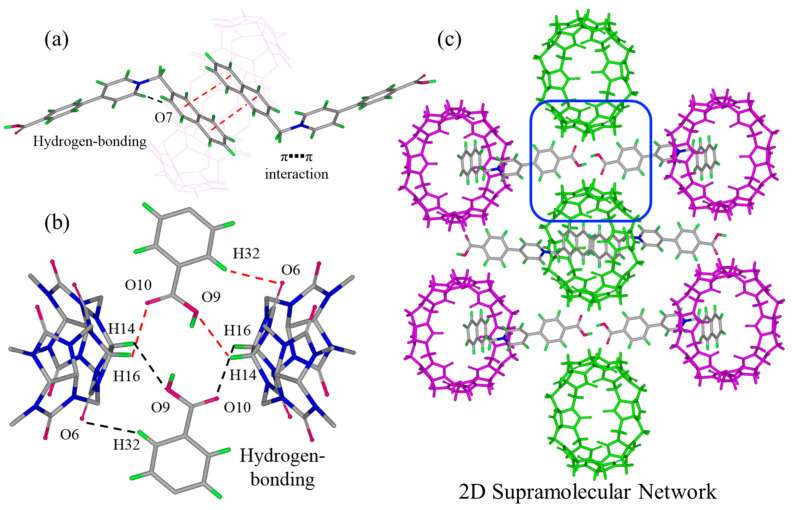
(**a**) X-ray crystal structure of the homoternary complex **NapA**_2_@Q[8]. Solvate water molecules were omitted for clarity. (**b**) The strong hydrogen-bonding between **NapA** and Q[8] molecules. (**c**) Two-dimensional supramolecular network of complexes.

X-ray structure analysis provided unequivocal proof of the formation of homoternary complexes between Q[8] and both **Nap1** and **NapA**. Crystal of **NapA**_2_@Q[8] was grown by slow evaporation of a solution containing the host Q[8] and the guest **NapA** under 3.0 M aqueous hydrochloric acid solution. X-ray structural analysis previously established that the **NapA**_2_@Q[8] crystallized in the orthorhombic crystal system, space group Pbca. As can be seen in Figure 5a, the naphthyl moiety of the **NapA** guest located inside the cavity of the Q[8] host, which was in agreement with what we had observed in the aqueous solution by ^1^H NMR spectroscopy. Furthermore, the π···π interactions between two encapsulated **NapA** molecules played a critical role in the formation of this host-guest inclusion complex. Obviously, the van der Waals contacted between the naphthyl groups and the inner wall of the Q[8] cavity, and strong hydrogen-bonding, such as C(32)-H···O(6) 2.701 Å (between carbonyl oxygen of host and H on benzene ring of guest), contributed to the formation of the inclusion complex **NapA**_2_@Q[8]. As shown in Figure 5b, two adjacent complexes formed a two dimensional assembled host–guest supramolecular network via hydrogen-bonding interactions between the portal carbonyl oxygen atom O6 of Q[8] and between the carboxyl oxygen atom O9, O10 of **NapA**. It is should be noted that the hydrogen bonds between the carboxyl oxygen atom of the guest **NapA** and the hydrogen atom on methylene of Q[8] (Figure 5b): C(14)-H···O(9) 2.571 Å, C(16)-H···O(10) 2.430 Å, and between the carbonyl oxygens at the portals of the Q[8] host and the hydrogen atom on the methylene of the adjacent Q[8] (Figure S4): C(11)-H···O(8) 2.696 Å may be largely responsible for the construction of a one-dimensional supramolecular chain (Figure S5) and supramolecular networks (Figure 5c).

**Figure 6:**
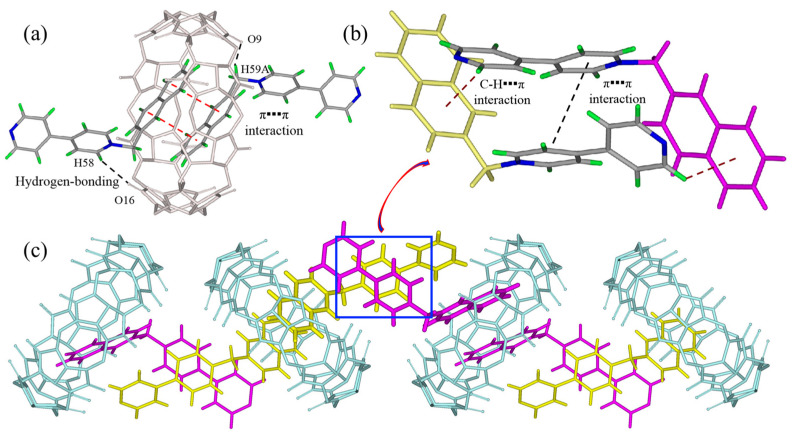
(**a**) X-ray crystal structure of the homoternary complex **Nap1**_2_@Q[8]. Solvate water molecules and [CdCl_4_]^2−^ anions were omitted for clarity. (**b**) The C–H···π interaction and π···π interaction between two neighboring **Nap1** molecules. (**c**) Crystal structure of a one-dimensional supramolecular chain constructed of the **Nap1** and Q[8].

Single crystals of complex **Nap1_2_**@Q[8] were fortunately obtained from hydrochloride acid solution by slow evaporation in the presence of CdCl_2_. The complex crystallized in the triclinic crystal system, space group P-1, the compounds consists of one Q[8] host and two **Nap1** guests. As shown in Figure 6a, X-ray structural analysis revealed that the naphthyl moiety of the guest **Nap1** adopted reverse parallel in the Q[8] cavity, the bipyridyl moiety remained outside of its portal, which could be attributed to π···π interactions in the cavity of Q[8]. Outside of the inclusion complexes, neighboring **Nap1** molecules contacted with each other through not only π···π interaction, but also C-H···π interactions (Figure 6b), which may serve to stabilize this structure for building a one-dimensional supramolecular chain of the complexes. In the latter, the encapsulated guests were electron donor and acceptor pair, and the major driving force for the ternary complex formation appears to be strong charge-transfer interaction between the guests [45]. Furthermore, the strong hydrogen-bonding played a critical role in the formation of this host-guest inclusion complex, e.g., between H on methylene of **Nap1** and the carbonyl oxygens at the portals of the Q[8] host C(59)–H···O(6) 2.691 Å, between H on pyridine ring and the carbonyl oxygens at the portals of the adjacent Q[8] host C(70)–H···O(10) 2.660 Å. Meanwhile, the hydrogen-bonding between two adjacent Q[8]: C(58)–H···O(16) 2.311 Å (Figure S6) and between [CdCl_4_]^2−^ and **Nap1**: C(23)–H···Cl(9) 2.692 Å, between [CdCl_4_]^2−^ and Q[8] C(53)–H···Cl(11) 2.836 Å (Figure S7) cannot be ignored in the construction of one-dimensional supramolecular chains (Figure 6c).

## 3. Materials and Methods

All reagents were obtained from commercial suppliers and used without further purification unless otherwise noted. All reactions were carried out under a dry nitrogen atmosphere. All solvents were dried before use following standard procedures. ^1^H and ^13^C NMR spectra were recorded on 400 MHz spectrometers in the indicated solvents at room temperature (298 K). Dynamic light scattering (DLS) measurement was conducted on Malvern Zetasizer Nano ZS90 using a monochromatic coherent He–Ne laser (633 nm) as the light source and a detector that detected the scattered light at an angle of 90°. An isothermal titration calorimetry (ITC) experiment was carried out using a MicroCal PEAQ-ITC (Malven Panalytical, Worcestershire, UK) instrument. Association constants and associated thermodynamic parameters were obtained through computer simulations (curve fitting) using Micro-Cal ITC analyze software (MicroCal Origin 4.1). UV–vis spectra were detected on a PerkinElmer 750s instrument from 200 to 800 nm at the scan rate of 3 nm/internal.

Single crystals of **NapA**_2_@Q[8] and **Nap1**_2_@Q[8] were grown from hydrochloride acid solution by slow evaporation. The crystal culture conditions showed that the self-assembly **NapA**_2_@Q[8] and **Nap1**_2_@Q[8] had good stability in strong acid solution. Diffraction data of both complexes were collected at 273(2) K with a Bruker SMART Apex-II CCD diffractometer using graphite-monochromated Mo-*K*α radiation (λ = 0.71073 Å). Empirical absorption corrections were performed by using the multi-scan program SADABS. Structural solution and full-matrix least-squares refinement based on *F2* were performed with the SHELXS-97 and SHELXL-97 program packages, respectively. Non-hydrogen atoms were treated anisotropically in all cases. All hydrogen atoms were introduced as riding atoms with an isotropic displacement parameter equal to 1.2 times that of the parent atom. Hydrogen atoms were given for all isolated water molecules.

CCDC 2223335 (**Nap1**_2_@Q[8]) and 2223329 (**NapA**_2_@Q[8]) contain the supplementary crystallographic data for this paper. These data can be obtained free of charge from The Cambridge Crystallographic Data Centre via www.ccdc.cam.ac.uk/data_request/cif, which accessed on 3 December 2022.

## 4. Conclusions

In summary, we investigated the host–guest complexation of Q[8] with two enantiomers, **NapA** and **Nap1**, in both aqueous solution and solid state by using NMR, UV-vis spectrum, ITC, DLS, and single-crystal X-ray crystallography. Driven by the cooperativity of electrostatic interactions, multiple C–H···π interactions, and hydrogen-bonding, both **NapA** and **Nap1** can be encapsulated into the cavity of Q[8] to form stable homoternary complexes **NapA**_2_@Q[8] and **Nap1**_2_@Q [8]. Structure analysis shows that hydrogen-bonding interactions and π···π interactions play a critical role not only in the formation of 1D extended chains, but also in the construction of 2D and 3D networks. This study shows that Q[8] host and molecules containing naphthalene units can be used as building units to build a diverse supramolecular network structure.

## Data Availability

No applicable.

## Supplementary Materials

The following supporting information can be downloaded at: https://www.mdpi.com/article/10.3390/molecules28010063/s1, Figure S1: ^1^H NMR spectrum (400 MHz) of the mixtures of **NapA** (1.0 mM) and Q[8] (1:0.5) in D_2_O at 25 °C; Figures S2 and S3: Job’s plot obtained from the absorption spectra of the mixtures of **NapA** or **Nap1** and Q[8] ([**NapA** or **Nap1**] + Q[8] = 50 μM) in water at 25 °C; Figures S4 and S5: Details of **NapA**_2_@Q[8] crystal structure; Figures S6 and S7: Details of **Nap1**_2_@Q[8] crystal structure; Figures S8–S11: Characterization of **NapA**; Figures S12–S15: Characterization of **Nap1**; Table S1. ITC measurements of the thermodynamics of **NapA**_2_@Q[8] and **Nap1**_2_@Q[8] interactions in aqueous solution at 298.15 K.

## Sample Availability

Samples of the compounds are not available from the authors.
