# Supplementary material for: Supramolecular Crystal Networks Constructed from Cucurbit[8]uril with Two Naphthyl Groups
Source: Molecules. 2022 Dec 21;28(1):63. doi: 10.3390/molecules28010063 (PMC9822147; doi:10.3390/molecules28010063)

checkCIF/PLATON report

Structure factors have been supplied for datablock(s) ga_80425a_a_sq

THIS REPORT IS FOR GUIDANCE ONLY. IF USED AS PART OF A REVIEW PROCEDURE FOR PUBLICATION, IT SHOULD NOT REPLACE THE EXPERTISE OF AN EXPERIENCED CRYSTALLOGRAPHIC REFEREE.

No syntax errors found. CIF dictionary Interpreting this report

Datablock: ga_80425a_a_sq

Bond precision: C-C = 0.0076 A Wavelength=1.34138

Cell:	a=18.3185(6)	b=18.3357(5)	c=19.5491(6)
	alpha=106.191(1)	beta=92.557(1)	gamma=97.633(1)
Temperature:	170 K		

	Calculated	Reported
Volume	6226.9(3)	6226.9(3)
Space group	P -1	P -1
Hall group	-P 1	-P 1
Moiety formula	2(C48 H48 N32 O16), 4(C21 H17 N2), 2(Cd2 Cl7), 2(Cd Cl4), Cl, 2	?
Sum formula	C180 H164 Cd6 Cl23 N72 O54 [+ solvent]	C90 H107.50 Cd3 Cl11.50 N36 O27
Mr	5689.64	2870.49
Dx,g cm-3	1.517	1.531
Z	1	2
Mu (mm-1)	4.731	4.732
F000	2859.0	2910.0
F000’	2873.17	
h,k,lmax	22,22,24	22,22,24
Nref	25482	25389
Tmin,Tmax	0.706,0.753	0.617,0.772
Tmin’	0.566	

Correction method= # Reported T Limits: Tmin=0.617 Tmax=0.772
AbsCorr = MULTI-SCAN

Data completeness= 0.996 Theta(max)= 56.976

R(reflections)= 0.0546(19734) wR2(reflections)= 0.1586(25389)

S = 1.042 Npar= 1689

The following ALERTS were generated. Each ALERT has the format
test-name_ALERT_alert-type_alert-level.
Click on the hyperlinks for more details of the test.

Alert level A

```
PLAT430_ALERT_2_A Short Inter D...A Contact  O20      ..O26             2.50 Ang.
                                                 x,y,z  =      1_555 Check
PLAT430_ALERT_2_A Short Inter D...A Contact  O21      ..O23             2.49 Ang.
                                                 x,y,z  =      1_555 Check
PLAT430_ALERT_2_A Short Inter D...A Contact  O22      ..O25             2.07 Ang.
                                                 x,y,z  =      1_555 Check
PLAT430_ALERT_2_A Short Inter D...A Contact  O23      ..O25             2.39 Ang.
                                           1-x,1-y,1-z  =      2_666 Check
```

Alert level B

```
PLAT306_ALERT_2_B Isolated Oxygen Atom (H-atoms Missing ?) .......        O19 Check
PLAT306_ALERT_2_B Isolated Oxygen Atom (H-atoms Missing ?) .......        O20 Check
PLAT306_ALERT_2_B Isolated Oxygen Atom (H-atoms Missing ?) .......        O21 Check
PLAT306_ALERT_2_B Isolated Oxygen Atom (H-atoms Missing ?) .......        O22 Check
PLAT306_ALERT_2_B Isolated Oxygen Atom (H-atoms Missing ?) .......        O23 Check
PLAT306_ALERT_2_B Isolated Oxygen Atom (H-atoms Missing ?) .......        O24 Check
PLAT306_ALERT_2_B Isolated Oxygen Atom (H-atoms Missing ?) .......        O25 Check
PLAT306_ALERT_2_B Isolated Oxygen Atom (H-atoms Missing ?) .......        O26 Check
PLAT306_ALERT_2_B Isolated Oxygen Atom (H-atoms Missing ?) .......        O27 Check
PLAT430_ALERT_2_B Short Inter D...A Contact  O1       ..O23             2.73 Ang.
                                               x,y,1+z  =      1_556 Check
PLAT430_ALERT_2_B Short Inter D...A Contact  O2       ..O20             2.73 Ang.
                                            -1+x,y,1+z  =      1_456 Check
PLAT430_ALERT_2_B Short Inter D...A Contact  O4       ..O24             2.76 Ang.
                                            -1+x,y,1+z  =      1_456 Check
PLAT430_ALERT_2_B Short Inter D...A Contact  O5       ..O19             2.82 Ang.
                                                 x,y,z  =      1_555 Check
PLAT430_ALERT_2_B Short Inter D...A Contact  O7       ..N33             2.65 Ang.
                                               x,y,1+z  =      1_556 Check
PLAT430_ALERT_2_B Short Inter D...A Contact  O11      ..O27             2.76 Ang.
                                                 x,y,z  =      1_555 Check
PLAT430_ALERT_2_B Short Inter D...A Contact  O15      ..O17             2.74 Ang.
                                              x,-1+y,z  =      1_545 Check
PLAT430_ALERT_2_B Short Inter D...A Contact  O17      ..N35             2.66 Ang.
                                                 x,y,z  =      1_555 Check
PLAT430_ALERT_2_B Short Inter D...A Contact  O20      ..O24             2.72 Ang.
                                                 x,y,z  =      1_555 Check
PLAT430_ALERT_2_B Short Inter D...A Contact  N35      ..O17'            2.79 Ang.
                                                 x,y,z  =      1_555 Check
PLAT934_ALERT_3_B Number of (Iobs-Icalc)/SigmaW > 10 Outliers ....          2 Check
```

Alert level C

```
PLAT230_ALERT_2_C Hirshfeld Test Diff for   O6       --C15       .        5.7 s.u.
PLAT241_ALERT_2_C High   'MainMol' Ueq as Compared to Neighbors of        C53 Check
PLAT244_ALERT_4_C Low    'Solvent' Ueq as Compared to Neighbors of        Cd3 Check
PLAT906_ALERT_3_C Large K Value in the Analysis of Variance ......      2.048 Check
PLAT911_ALERT_3_C Missing FCF Refl Between Thmin & STh/L=    0.600         24 Report
PLAT975_ALERT_2_C Check Calcd Resid. Dens.  1.00A   From O24            0.61 eA-3
PLAT975_ALERT_2_C Check Calcd Resid. Dens.  1.04A   From O20            0.61 eA-3
PLAT976_ALERT_2_C Check Calcd Resid. Dens.  0.52A   From O23           -0.94 eA-3
PLAT976_ALERT_2_C Check Calcd Resid. Dens.  1.04A   From O23           -0.66 eA-3
PLAT976_ALERT_2_C Check Calcd Resid. Dens.  1.01A   From O22           -0.63 eA-3
```

```
PLAT976_ALERT_2_C Check Calcd Resid. Dens.  0.47A   From O26           -0.59 eA-3
PLAT976_ALERT_2_C Check Calcd Resid. Dens.  0.41A   From O25           -0.51 eA-3
PLAT976_ALERT_2_C Check Calcd Resid. Dens.  0.50A   From O22           -0.47 eA-3
PLAT976_ALERT_2_C Check Calcd Resid. Dens.  0.60A   From O21           -0.46 eA-3
PLAT976_ALERT_2_C Check Calcd Resid. Dens.  0.52A   From O21           -0.44 eA-3
PLAT976_ALERT_2_C Check Calcd Resid. Dens.  0.64A   From O22           -0.43 eA-3
PLAT978_ALERT_2_C Number C-C Bonds with Positive Residual Density.         0 Info
```

Alert level G

```
FORMU01_ALERT_2_G  There is a discrepancy between the atom counts in the
            _chemical_formula_sum and the formula from the _atom_site* data.
            Atom count from _chemical_formula_sum:C90 H107.5 Cd3 Cl11.5 N36 O27
            Atom count from the _atom_site data:  C90. H82 Cd3 Cl11.5 N36 O27
ABSMU01_ALERT_1_G  Calculation of _exptl_absorpt_correction_mu
                not performed for this radiation type.
CELLZ01_ALERT_1_G Difference between formula and atom_site contents detected.
CELLZ01_ALERT_1_G WARNING: H atoms missing from atom site list. Is this intentional?
           From the CIF: _cell_formula_units_Z    2
           From the CIF: _chemical_formula_sum  C90 H107.50 Cd3 Cl11.50 N36 O27
           TEST: Compare cell contents of formula and atom_site data

           atom    Z*formula  cif sites diff
           C        180.00    180.00    0.00
           H        215.00    164.00   51.00
           Cd         6.00      6.00    0.00
           Cl        23.00     23.00    0.00
           N         72.00     72.00    0.00
           O         54.00     54.00    0.00
PLAT002_ALERT_2_G Number of Distance or Angle Restraints on AtSite         19 Note
PLAT003_ALERT_2_G Number of Uiso or Uij Restrained non-H Atoms ...         49 Report
PLAT041_ALERT_1_G Calc. and Reported SumFormula    Strings  Differ     Please Check
PLAT045_ALERT_1_G Calculated and Reported Z Differ by a Factor ...       0.50 Check
PLAT068_ALERT_1_G Reported F000 Differs from Calcd (or Missing)...     Please Check
PLAT083_ALERT_2_G SHELXL Second Parameter in WGHT  Unusually Large      12.85 Why ?
PLAT154_ALERT_1_G The s.u.'s on the Cell Angles are Equal ..(Note)      0.001 Degree
PLAT171_ALERT_4_G The CIF-Embedded .res File Contains EADP Records          2 Report
PLAT172_ALERT_4_G The CIF-Embedded .res File Contains DFIX Records          2 Report
PLAT178_ALERT_4_G The CIF-Embedded .res File Contains SIMU Records          2 Report
PLAT186_ALERT_4_G The CIF-Embedded .res File Contains ISOR Records          5 Report
PLAT233_ALERT_4_G Hirshfeld (M-X Solvent)   Cd2      --Cl4       .       10.8 s.u.
PLAT302_ALERT_4_G Anion/Solvent/Minor-Residue Disorder (Resd  3  )        48% Note
PLAT302_ALERT_4_G Anion/Solvent/Minor-Residue Disorder (Resd  4  )        48% Note
PLAT302_ALERT_4_G Anion/Solvent/Minor-Residue Disorder (Resd  7  )       100% Note
PLAT302_ALERT_4_G Anion/Solvent/Minor-Residue Disorder (Resd  8  )       100% Note
PLAT302_ALERT_4_G Anion/Solvent/Minor-Residue Disorder (Resd  9  )       100% Note
PLAT302_ALERT_4_G Anion/Solvent/Minor-Residue Disorder (Resd 10  )       100% Note
PLAT302_ALERT_4_G Anion/Solvent/Minor-Residue Disorder (Resd 20  )       100% Note
PLAT302_ALERT_4_G Anion/Solvent/Minor-Residue Disorder (Resd 21  )       100% Note
PLAT304_ALERT_4_G Non-Integer Number of Atoms in ...... Resd  7          0.35 Check
PLAT304_ALERT_4_G Non-Integer Number of Atoms in ...... Resd  8          0.15 Check
PLAT304_ALERT_4_G Non-Integer Number of Atoms in ...... Resd  9          0.80 Check
PLAT304_ALERT_4_G Non-Integer Number of Atoms in ...... Resd 10          0.51 Check
PLAT304_ALERT_4_G Non-Integer Number of Atoms in ...... Resd 20          0.20 Check
PLAT304_ALERT_4_G Non-Integer Number of Atoms in ...... Resd 21          0.49 Check
PLAT311_ALERT_2_G Isolated Disordered Oxygen Atom (No H's ?) .....        O17 Check
PLAT311_ALERT_2_G Isolated Disordered Oxygen Atom (No H's ?) .....       O18' Check
PLAT311_ALERT_2_G Isolated Disordered Oxygen Atom (No H's ?) .....       O17' Check
PLAT311_ALERT_2_G Isolated Disordered Oxygen Atom (No H's ?) .....        O18 Check
PLAT431_ALERT_2_G Short Inter HL..A Contact  Cl10     ..O21              3.12 Ang.
                                                     x,y,z  =      1_555 Check
```

```
PLAT431_ALERT_2_G Short Inter HL..A Contact  Cl11     ..O22              3.12 Ang.
                                                    x,y,z  =      1_555 Check
PLAT432_ALERT_2_G Short Inter X...Y Contact  Cl10     ..C19              3.11 Ang.
                                                    x,y,z  =      1_555 Check
PLAT432_ALERT_2_G Short Inter X...Y Contact  O3       ..C90'             2.96 Ang.
                                                  x,y,1+z  =      1_556 Check
PLAT432_ALERT_2_G Short Inter X...Y Contact  O7       ..C49              2.99 Ang.
                                                  x,y,1+z  =      1_556 Check
PLAT432_ALERT_2_G Short Inter X...Y Contact  O10      ..C70              3.01 Ang.
                                              1-x,1-y,1-z  =      2_666 Check
PLAT432_ALERT_2_G Short Inter X...Y Contact  O12      ..C76              3.01 Ang.
                                              1-x,1-y,1-z  =      2_666 Check
PLAT432_ALERT_2_G Short Inter X...Y Contact  O14      ..C69'             2.95 Ang.
                                              1-x,1-y,1-z  =      2_666 Check
PLAT432_ALERT_2_G Short Inter X...Y Contact  O22      ..C77              2.98 Ang.
                                              1-x,1-y,1-z  =      2_666 Check
PLAT432_ALERT_2_G Short Inter X...Y Contact  O22      ..C71              3.01 Ang.
                                              1-x,1-y,1-z  =      2_666 Check
PLAT606_ALERT_4_G VERY LARGE Solvent Accessible VOID(S) in Structure        ! Info
PLAT794_ALERT_5_G Tentative Bond Valency for Cd1       (II)      .       2.07 Info
PLAT794_ALERT_5_G Tentative Bond Valency for Cd2       (II)      .       1.99 Info
PLAT794_ALERT_5_G Tentative Bond Valency for Cd3       (II)      .       1.94 Info
PLAT860_ALERT_3_G Number of Least-Squares Restraints .............        402 Note
PLAT869_ALERT_4_G ALERTS Related to the Use of SQUEEZE Suppressed           ! Info
PLAT910_ALERT_3_G Missing # of FCF Reflection(s) Below Theta(Min).          2 Note
PLAT912_ALERT_4_G Missing # of FCF Reflections Above STh/L=  0.600         67 Note
PLAT933_ALERT_2_G Number of OMIT Records in Embedded .res File ...          1 Note
```

---

4 **ALERT level A** = Most likely a serious problem - resolve or explain
20 **ALERT level B** = A potentially serious problem, consider carefully
17 **ALERT level C** = Check. Ensure it is not caused by an omission or oversight
53 **ALERT level G** = General information/check it is not something unexpected

7 ALERT type 1 CIF construction/syntax error, inconsistent or missing data
56 ALERT type 2 Indicator that the structure model may be wrong or deficient
5 ALERT type 3 Indicator that the structure quality may be low
23 ALERT type 4 Improvement, methodology, query or suggestion
3 ALERT type 5 Informative message, check

It is advisable to attempt to resolve as many as possible of the alerts in all categories. Often the minor alerts point to easily fixed oversights, errors and omissions in your CIF or refinement strategy, so attention to these fine details can be worthwhile. In order to resolve some of the more serious problems it may be necessary to carry out additional measurements or structure refinements. However, the purpose of your study may justify the reported deviations and the more serious of these should normally be commented upon in the discussion or experimental section of a paper or in the "special_details" fields of the CIF. checkCIF was carefully designed to identify outliers and unusual parameters, but every test has its limitations and alerts that are not important in a particular case may appear. Conversely, the absence of alerts does not guarantee there are no aspects of the results needing attention. It is up to the individual to critically assess their own results and, if necessary, seek expert advice.

Publication of your CIF in IUCr journals

A basic structural check has been run on your CIF. These basic checks will be run on all CIFs submitted for publication in IUCr journals (*Acta Crystallographica*, *Journal of Applied Crystallography*, *Journal of Synchrotron Radiation*); however, if you intend to submit to *Acta Crystallographica Section C* or *E* or *IUCrData*, you should make sure that full publication checks are run on the final version of your CIF prior to submission.

Publication of your CIF in other journals

Please refer to the *Notes for Authors* of the relevant journal for any special instructions relating to CIF submission.

PLATON version of 14/07/2018; check.def file version of 05/06/2018

Datablock ga_80425a_a_sq - ellipsoid plot

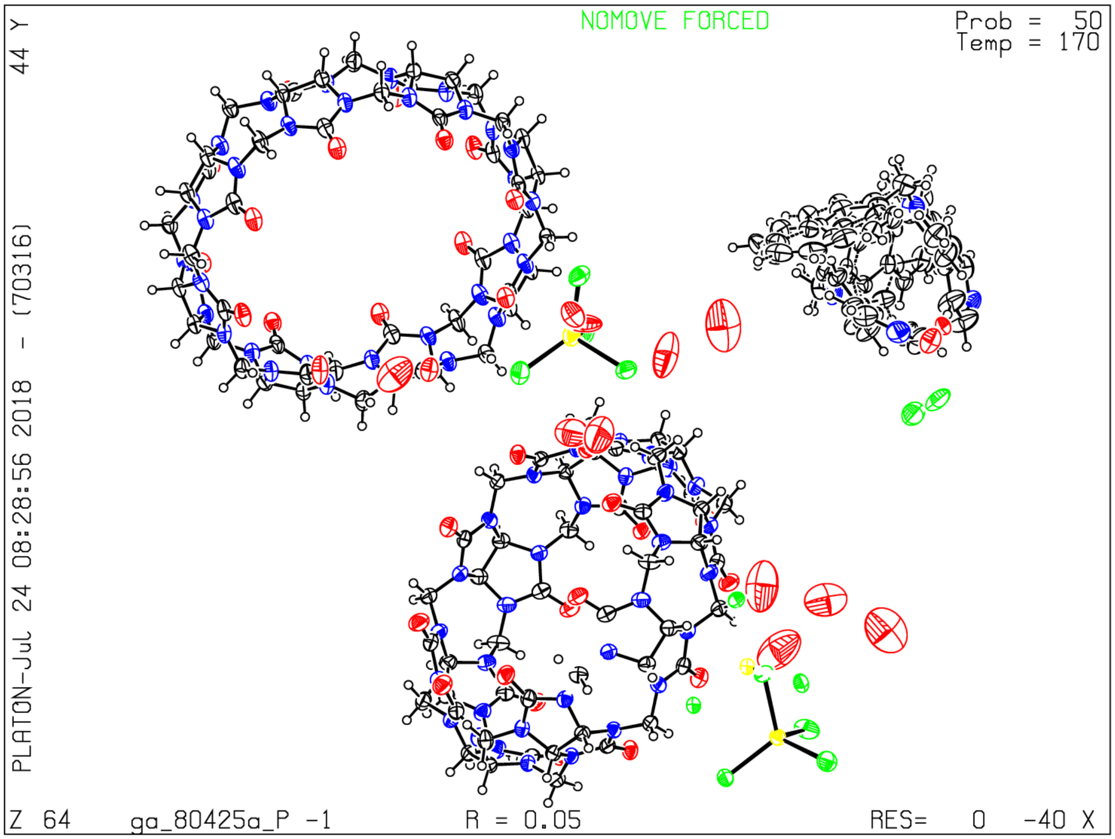

Supplement: Supplementary file 1 [file molecules-28-00063-s001.zip › molecules-2104080-supplementary/Supporting information/Nap1 checkcif.pdf]
