# Supplementary material for: Supramolecular Crystal Networks Constructed from Cucurbit[8]uril with Two Naphthyl Groups
Source: Molecules. 2022 Dec 21;28(1):63. doi: 10.3390/molecules28010063 (PMC9822147; doi:10.3390/molecules28010063)

checkCIF/PLATON report

Structure factors have been supplied for datablock(s) platon_sq

THIS REPORT IS FOR GUIDANCE ONLY. IF USED AS PART OF A REVIEW PROCEDURE FOR PUBLICATION, IT SHOULD NOT REPLACE THE EXPERTISE OF AN EXPERIENCED CRYSTALLOGRAPHIC REFEREE.

No syntax errors found.   CIF dictionary   Interpreting this report

Datablock: platon_sq

Bond precision:	C-C = 0.0082 A		Wavelength=1.34138
Cell:	a=19.2256(6)	b=21.7208(7)	c=30.3651(9)
	alpha=90	beta=90	gamma=90
Temperature:	173 K		

	Calculated	Reported
Volume	12680.3(7)	12680.3(7)
Space group	P b c a	P b c a
Hall group	: -P 2ac 2a	-P 2ac 2a
Moiety formula	C48 H48 N32 O16, 2(C23 H18 N O2), 4(Cl), 12(O) [+ solvent]	?
Sum formula	C94 H84 Cl4 N34 O32 [+ solvent]	C94 H110 Cl4 N34 O32
Mr	2343.75	2369.95
Dx,g cm-3	1.228	1.241
Z	4	4
Mu (mm-1)	1.009	1.010
F000	4840.0	4944.0
F000’	4857.56	
h,k,lmax	23,26,37	23,26,37
Nref	12047	11990
Tmin,Tmax	0.712,0.724	0.465,0.751
Tmin’	0.636	

Correction method= # Reported T Limits: Tmin=0.465 Tmax=0.751
AbsCorr = MULTI-SCAN

Data completeness= 0.995   Theta(max)= 54.856

R(reflections)= 0.1028(9628)   wR2(reflections)= 0.3507(11990)

S = 1.210   Npar= 749

The following ALERTS were generated. Each ALERT has the format
test-name_ALERT_alert-type_alert-level.
Click on the hyperlinks for more details of the test.

Alert level A

```
PLAT260_ALERT_2_A Large Average Ueq of Residue Including       O16      0.208 Check
PLAT430_ALERT_2_A Short Inter D...A Contact  O11      ..O13      .       2.51 Ang.
                                                      x,y,z  =      1_555 Check
```

Alert level B

```
PLAT260_ALERT_2_B Large Average Ueq of Residue Including      CL2'      0.189 Check
PLAT306_ALERT_2_B Isolated Oxygen Atom (H-atoms Missing ?) .......        O11 Check
PLAT306_ALERT_2_B Isolated Oxygen Atom (H-atoms Missing ?) .......        O12 Check
PLAT306_ALERT_2_B Isolated Oxygen Atom (H-atoms Missing ?) .......        O13 Check
PLAT306_ALERT_2_B Isolated Oxygen Atom (H-atoms Missing ?) .......        O14 Check
PLAT306_ALERT_2_B Isolated Oxygen Atom (H-atoms Missing ?) .......        O15 Check
PLAT306_ALERT_2_B Isolated Oxygen Atom (H-atoms Missing ?) .......        O16 Check
PLAT430_ALERT_2_B Short Inter D...A Contact  O2       ..O11      .       2.73 Ang.
                                              1/2-x,1/2+y,z  =      8_665 Check
PLAT430_ALERT_2_B Short Inter D...A Contact  O4       ..O11      .       2.80 Ang.
                                              1/2-x,1/2+y,z  =      8_665 Check
PLAT430_ALERT_2_B Short Inter D...A Contact  O10      ..N9       .       2.86 Ang.
                                             3/2-x,-1/2+y,z  =      8_755 Check
PLAT430_ALERT_2_B Short Inter D...A Contact  O13      ..O15      .       2.60 Ang.
                                                      x,y,z  =      1_555 Check
PLAT430_ALERT_2_B Short Inter D...A Contact  O13      ..O14      .       2.67 Ang.
                                             x,1/2-y,-1/2+z  =      7_565 Check
PLAT934_ALERT_3_B Number of (Iobs-Icalc)/SigmaW > 10 Outliers ....          6 Check
```

Alert level C

```
PLAT084_ALERT_3_C High wR2 Value (i.e. > 0.25) ...................       0.35 Report
PLAT112_ALERT_2_C ADDSYM Detects New (Pseudo) Symm. Elem         C         80 %Fit
PLAT230_ALERT_2_C Hirshfeld Test Diff for    C37      --C46      .        5.8 s.u.
PLAT234_ALERT_4_C Large Hirshfeld Difference C27      --C28      .       0.16 Ang.
PLAT234_ALERT_4_C Large Hirshfeld Difference C42      --C43      .       0.18 Ang.
PLAT234_ALERT_4_C Large Hirshfeld Difference C43      --C44      .       0.17 Ang.
PLAT241_ALERT_2_C High   'MainMol' Ueq as Compared to Neighbors of        C36 Check
PLAT241_ALERT_2_C High   'MainMol' Ueq as Compared to Neighbors of        C43 Check
PLAT241_ALERT_2_C High   'MainMol' Ueq as Compared to Neighbors of        C46 Check
PLAT242_ALERT_2_C Low    'MainMol' Ueq as Compared to Neighbors of        C37 Check
PLAT242_ALERT_2_C Low    'MainMol' Ueq as Compared to Neighbors of        C44 Check
PLAT260_ALERT_2_C Large Average Ueq of Residue Including       CL2      0.127 Check
PLAT260_ALERT_2_C Large Average Ueq of Residue Including       O11      0.102 Check
PLAT260_ALERT_2_C Large Average Ueq of Residue Including       O12      0.138 Check
PLAT260_ALERT_2_C Large Average Ueq of Residue Including       O13      0.120 Check
PLAT260_ALERT_2_C Large Average Ueq of Residue Including       O14      0.128 Check
PLAT260_ALERT_2_C Large Average Ueq of Residue Including       O15      0.133 Check
PLAT334_ALERT_2_C Small Aver. Benzene C-C Dist C39      -C44             1.37 Ang.
PLAT340_ALERT_3_C Low Bond Precision on  C-C Bonds ...............    0.00818 Ang.
PLAT430_ALERT_2_C Short Inter D...A Contact  O7       ..O16      .       2.90 Ang.
                                              x,3/2-y,1/2+z  =      7_576 Check
PLAT906_ALERT_3_C Large K Value in the Analysis of Variance ......      9.117 Check
PLAT911_ALERT_3_C Missing FCF Refl Between Thmin & STh/L=    0.600         35 Report
PLAT913_ALERT_3_C Missing # of Very Strong Reflections in FCF ....          4 Note
PLAT918_ALERT_3_C Reflection(s) with I(obs) much Smaller I(calc) .          7 Check
PLAT975_ALERT_2_C Check Calcd Resid. Dens.  0.92A   From O16             0.67 eA-3
PLAT976_ALERT_2_C Check Calcd Resid. Dens.  0.99A   From O16            -0.46 eA-3
```

```
PLAT977_ALERT_2_C Check Negative Difference Density on H46            -0.36 eA-3
PLAT978_ALERT_2_C Number C-C Bonds with Positive Residual Density.         0 Info
```

Alert level G

```
FORMU01_ALERT_2_G  There is a discrepancy between the atom counts in the
            _chemical_formula_sum and the formula from the _atom_site* data.
            Atom count from _chemical_formula_sum:C94 H110 Cl4 N34 O32
            Atom count from the _atom_site data:  C94 H84 Cl4 N34 O32
ABSMU01_ALERT_1_G  Calculation of _exptl_absorpt_correction_mu
                not performed for this radiation type.
CELLZ01_ALERT_1_G Difference between formula and atom_site contents detected.
CELLZ01_ALERT_1_G WARNING: H atoms missing from atom site list. Is this intentional?
           From the CIF: _cell_formula_units_Z    4
           From the CIF: _chemical_formula_sum  C94 H110 Cl4 N34 O32
           TEST: Compare cell contents of formula and atom_site data

           atom    Z*formula  cif sites diff
           C        376.00    376.00    0.00
           H        440.00    336.00  104.00
           Cl        16.00     16.00    0.00
           N        136.00    136.00    0.00
           O        128.00    128.00    0.00
PLAT002_ALERT_2_G Number of Distance or Angle Restraints on AtSite          2 Note
PLAT003_ALERT_2_G Number of Uiso or Uij Restrained non-H Atoms ...          3 Report
PLAT007_ALERT_5_G Number of Unrefined Donor-H Atoms ..............          1 Report
PLAT041_ALERT_1_G Calc. and Reported SumFormula    Strings  Differ     Please Check
PLAT068_ALERT_1_G Reported F000 Differs from Calcd (or Missing)...     Please Check
PLAT072_ALERT_2_G SHELXL First  Parameter in WGHT  Unusually Large       0.20 Report
PLAT083_ALERT_2_G SHELXL Second Parameter in WGHT  Unusually Large      13.50 Why ?
PLAT172_ALERT_4_G The CIF-Embedded .res File Contains DFIX Records          1 Report
PLAT178_ALERT_4_G The CIF-Embedded .res File Contains SIMU Records          1 Report
PLAT186_ALERT_4_G The CIF-Embedded .res File Contains ISOR Records          1 Report
PLAT300_ALERT_4_G Atom Site Occupancy of Cl2        Constrained at        0.8 Check
PLAT300_ALERT_4_G Atom Site Occupancy of Cl2’       Constrained at        0.2 Check
PLAT302_ALERT_4_G Anion/Solvent/Minor-Residue Disorder (Resd  4  )       100% Note
PLAT302_ALERT_4_G Anion/Solvent/Minor-Residue Disorder (Resd  5  )       100% Note
PLAT304_ALERT_4_G Non-Integer Number of Atoms in ...... Resd  4          0.80 Check
PLAT304_ALERT_4_G Non-Integer Number of Atoms in ...... Resd  5          0.20 Check
PLAT335_ALERT_2_G Check Large C6 Ring C-C Range C37      -C46            0.15 Ang.
PLAT335_ALERT_2_G Check Large C6 Ring C-C Range C39      -C44            0.18 Ang.
PLAT431_ALERT_2_G Short Inter HL..A Contact  Cl1      ..O15      .       3.15 Ang.
                                              1/2+x,y,1/2-z  =      6_656 Check
PLAT432_ALERT_2_G Short Inter X...Y Contact  O1       ..C47              2.99 Ang.
                                                1-x,1-y,1-z  =      5_666 Check
PLAT432_ALERT_2_G Short Inter X...Y Contact  O10      ..C13              2.87 Ang.
                                             3/2-x,-1/2+y,z  =      8_755 Check
PLAT432_ALERT_2_G Short Inter X...Y Contact  O10      ..C16              2.89 Ang.
                                             3/2-x,-1/2+y,z  =      8_755 Check
PLAT606_ALERT_4_G VERY LARGE Solvent Accessible VOID(S) in Structure       ! Info
PLAT860_ALERT_3_G Number of Least-Squares Restraints ............         25 Note
PLAT869_ALERT_4_G ALERTS Related to the Use of SQUEEZE Suppressed          ! Info
PLAT910_ALERT_3_G Missing # of FCF Reflection(s) Below Theta(Min).         1 Note
PLAT912_ALERT_4_G Missing # of FCF Reflections Above STh/L=  0.600        21 Note
PLAT955_ALERT_1_G Reported (CIF) and Actual (FCF) Lmax Differ by .         1 Units
```

---

2 **ALERT level A** = Most likely a serious problem - resolve or explain
13 **ALERT level B** = A potentially serious problem, consider carefully
28 **ALERT level C** = Check. Ensure it is not caused by an omission or oversight
32 **ALERT level G** = General information/check it is not something unexpected

```
 6 ALERT type 1 CIF construction/syntax error, inconsistent or missing data
44 ALERT type 2 Indicator that the structure model may be wrong or deficient
 9 ALERT type 3 Indicator that the structure quality may be low
15 ALERT type 4 Improvement, methodology, query or suggestion
 1 ALERT type 5 Informative message, check
```

It is advisable to attempt to resolve as many as possible of the alerts in all categories. Often the minor alerts point to easily fixed oversights, errors and omissions in your CIF or refinement strategy, so attention to these fine details can be worthwhile. In order to resolve some of the more serious problems it may be necessary to carry out additional measurements or structure refinements. However, the purpose of your study may justify the reported deviations and the more serious of these should normally be commented upon in the discussion or experimental section of a paper or in the "special_details" fields of the CIF. checkCIF was carefully designed to identify outliers and unusual parameters, but every test has its limitations and alerts that are not important in a particular case may appear. Conversely, the absence of alerts does not guarantee there are no aspects of the results needing attention. It is up to the individual to critically assess their own results and, if necessary, seek expert advice.

Publication of your CIF in IUCr journals

A basic structural check has been run on your CIF. These basic checks will be run on all CIFs submitted for publication in IUCr journals (*Acta Crystallographica*, *Journal of Applied Crystallography*, *Journal of Synchrotron Radiation*); however, if you intend to submit to *Acta Crystallographica Section C* or *E* or *IUCrData*, you should make sure that full publication checks are run on the final version of your CIF prior to submission.

Publication of your CIF in other journals

Please refer to the *Notes for Authors* of the relevant journal for any special instructions relating to CIF submission.

PLATON version of 26/09/2018; check.def file version of 13/09/2018

Datablock platon_sq - ellipsoid plot

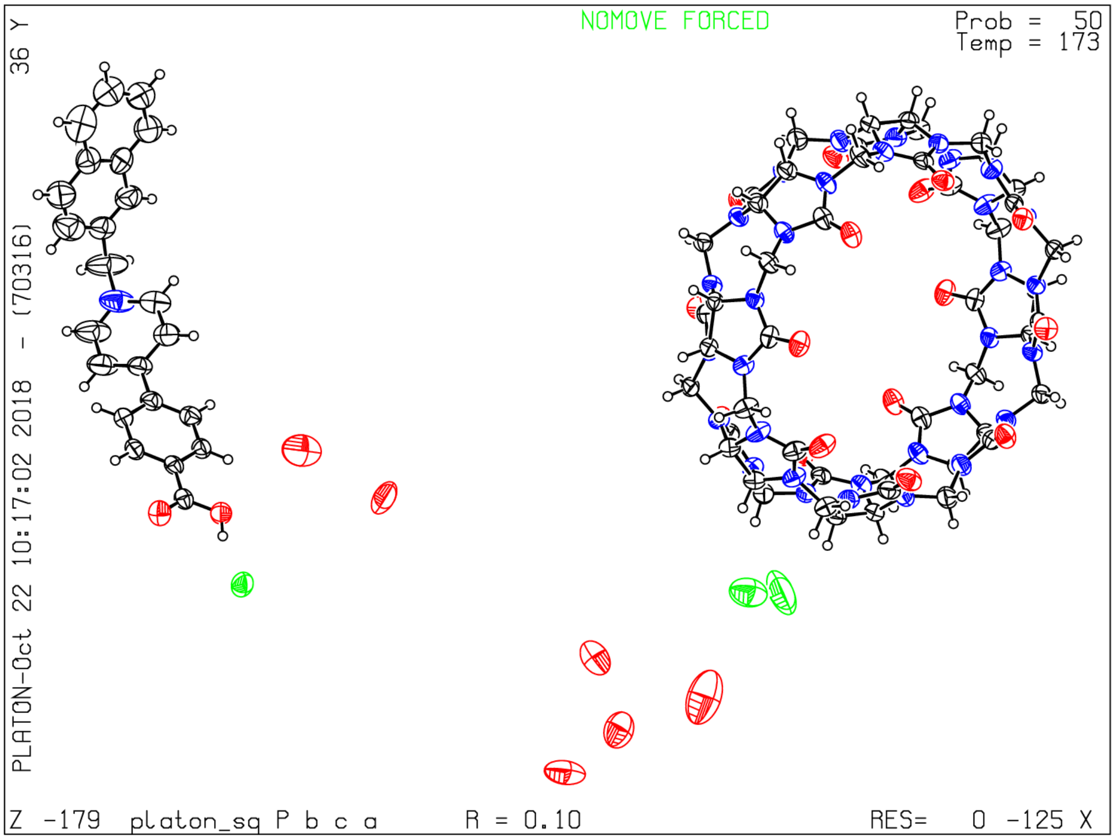

Supplement: Supplementary file 1 [file molecules-28-00063-s001.zip › molecules-2104080-supplementary/Supporting information/NapA checkcif.pdf]
