# Supplementary material for: Supramolecular Crystal Networks Constructed from Cucurbit[8]uril with Two Naphthyl Groups
Source: Molecules. 2022 Dec 21;28(1):63. doi: 10.3390/molecules28010063 (PMC9822147; doi:10.3390/molecules28010063)
Supplement: Supplementary file 1 [file molecules-28-00063-s001.zip › molecules-2104080-supplementary/Supporting information/Supporting information.docx]

Supporting information

**General Information.**

Deionized water was used for all experiments. Starting materials and solvents for syntheses were purchased commercially and used as supplied without further purification. Q[8] was prepared and purified according to our previously published procedure. 2-Bromomethyl naphthalen, 4-(4-pyridyl)benzoic acid, and 4,4'-bipyridine were received from Aldrich Chemical, USA and purified by a vacuum sublimation process.

^1^H NMR spectra was recorded by Bruker AVANCE III HD 400 MHz (100 MHz) instrument, and were internally referenced to residual solvent signals (^1^H NMR: D_2_O referenced at 4.67 ppm, DMSO-*d6* referenced at 2.54 ppm) at room temperature (298 K). An isothermal titration calorimetry (ITC) experiment was carried out using a MicroCal PEAQITC instrument. Association constants and associated thermodynamic parameters were obtained through computer simulations (curve fitting) using Micro-Cal ITC analyze software. UV-Vis absorption spectra of the host–guest complexes were recorded in 1 cm quartz cells on an Agilent 8453 spectrophotometer at room temperature. Dynamic light scattering (DLS) measurement was conducted on Malvern Zetasizer Nano ZS90 using a monochromatic coherent He−Ne laser (633 nm) as the light source and a detector that detected the scattered light at an angle of 90°.

Single crystals of **NapA**_2_@Q[8] and for **Nap1**_2_@Q[8] were grown from hydrochloride acid solution by slow evaporation. Diffraction data of both complexes were collected at 273(2) K with a Bruker SMART Apex-II CCD diffractometer using graphite-monochromated Mo-*K*α radiation (λ = 0.71073). Empirical absorption corrections were performed by using the multi-scan program SADABS. Structural solution and full-matrix least-squares refinement based on *F2* were performed with the SHELXS-97 and SHELXL-97 program packages, respectively. Non-hydrogen atoms were treated anisotropically in all cases. All hydrogen atoms were introduced as riding atoms with an isotropic displacement parameter equal to 1.2 times that of the parent atom. Hydrogen atoms were given for all isolated water molecules. CCDC 2223335 (**Nap1**_2_@Q[8]) and 2223329 (**NapA**_2_@Q[8]) contain the supplementary crystallographic data for this paper. These data can be obtained free of charge from The Cambridge Crystallographic Data Centre via www.ccdc.cam.ac.uk/data_request/cif.

**Synthesis of compounds**

**Compound NapA.** A mixture of compounds 2-bromomethyl naphthalen (0.46g, 2.1 mmol) and 4-(4-pyridyl)benzoic acid (0.40g, 2.0 mmol) in acetonitrile (20 mL) were stirred for 4 h at 75 °C and then cooled to room temperature. Ether (10 mL) was added to the mixture. The resulting precipitate was filtrated, then washed with ether (5 times) and dried in vacuum to give compound **NapA** as a white solid (0.75 g, 90%). M.p. > 300 °C (decomp). ^1^H NMR (400 MHz, DMSO-*d6*) : δ 13.40 (m, 1H), 9.37 (d, *J* = 7.1 Hz, 2H), 8.62 (d, *J* = 7.0 Hz, 2H), 8.18 (d, *J* = 8.8 Hz, 2H), 8.14 (d, *J* = 6.6 Hz, 3H), 8.01 (d, *J* = 8.6 Hz, 1H), 7.96 (t, *J* = 4.6 Hz, 2H), 7.69-7.66 (dd, *J* = 1.8 Hz, 8.5Hz, 1H), 7.59-7.56 (dd, *J* = 3.8 Hz, 6.1Hz, 2H), 6.06 (m, 2H). ^13^C NMR (100 MHz, DMSO-*d6*): δ 166.7, 154.2, 145.2, 137.5, 133.7, 133.0, 132.8, 131.9, 130.3, 129.1, 128.7, 128.4, 128.1, 127.8, 127.0, 125.9, 125.6, 62.9. HRMS (ESI): Calcd for C_23_H_18_NO_2_^+^: 340.1332[M-Br]^+^. Found: 340.1346.

**Compound Nap1.** Dissolve naphthalene (0.46g, 2.1 mmol) and 4,4'-bipyridine (0.40g, 2.0 mmol) in 10 mL acetone respectively, and then slowly drop naphthalene solution into bipyridine solution. The mixed solution were stirred for 1 h at 50 °C and then cooled to room temperature. Ether (10 mL) was added to the mixture. The resulting precipitate was filtrated and washed with ether (5 times) and dried in vacuum to give compound **Nap1** as a yellow solid (0.75 g, 85%). M.p. > 300 °C (decomp). ^1^H NMR (400 MHz, D_2_O) : δ 8.93 (d, *J =* 9.4 Hz*,* 2H), 8.65-8.63 (dd, *J* = 1.8 Hz, 4.6 Hz, 2H), 8.27 (d, *J* = 6.8 Hz, 2H), 7.94 (m, 2H), 7.88 (d, *J* = 8.6 Hz, 2H), 7.56 (dd, *J* = 4.7 Hz, 9.6 Hz, 2H), 7.76-7.75 (dd, *J* = 1.7 Hz, 4.6 Hz, 2H), 7.53-7.49 (dd, *J* = 3.2 Hz, *J* = 6.2 Hz, 2H) 7.44-7.41 (dd, *J* = 1.9 Hz, 8.6 Hz, 1H), 5.89 (m, 2H). ^13^C NMR (100 MHz, DMSO-*d6*): δ 153.2, 151.4, 145.9, 141.3, 133.4, 133.1, 132.2, 129.5, 128.9, 128.2, 127.6, 127.3, 126.3, 126.2, 122.4, 63.4. HRMS (ESI): Calcd for C_21_H_17_N_2_^+^: 297.1386, [M-Br]^+^. Found: 297.1382.


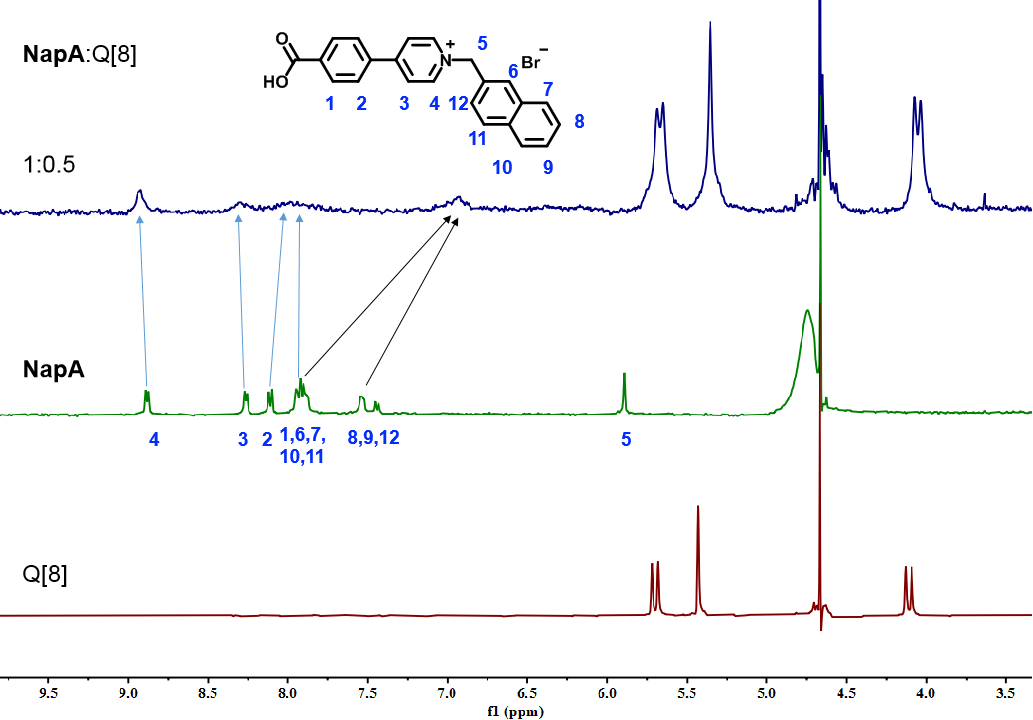


**Figure S1.** ^1^H NMR spectrum (400 MHz) of the mixtures of **NapA** (1.0 mM) and Q[8] (1:0.5) in D_2_O at 25 °C.

**Figure S2.** Job’s plot obtained from the absorption spectra of the mixtures of **NapA** and Q[8] ([**NapA**] + [Q[8]] = 50 μM) in water at 25 °C.

**Figure S3.** Job’s plot obtained from the absorption spectra of the mixtures of **Nap1** and Q[8] ([**Nap1**] + [Q[8]] = 50 μM) in water at 25 °C.


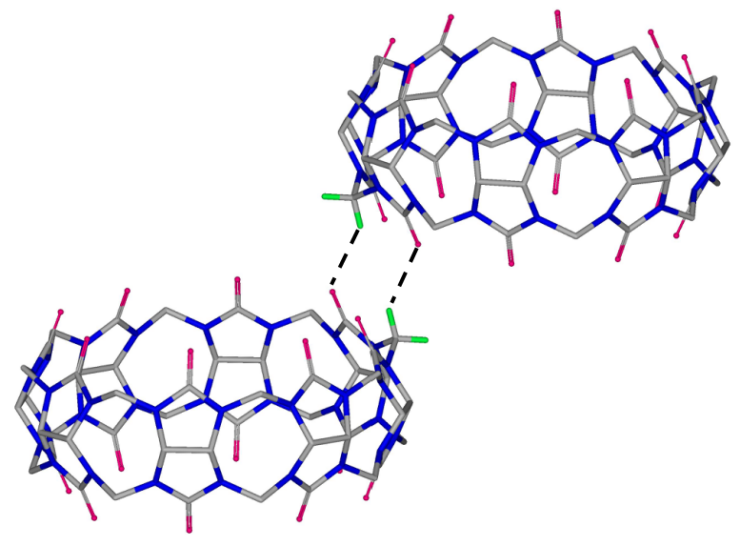


**Figure S4.** The hydrogen bonds between the carbonyl oxygens at the portals of the Q[8] host and the hydrogen atom on the methylene of the adjacent Q[8] (**NapA_2_**@Q[8]).


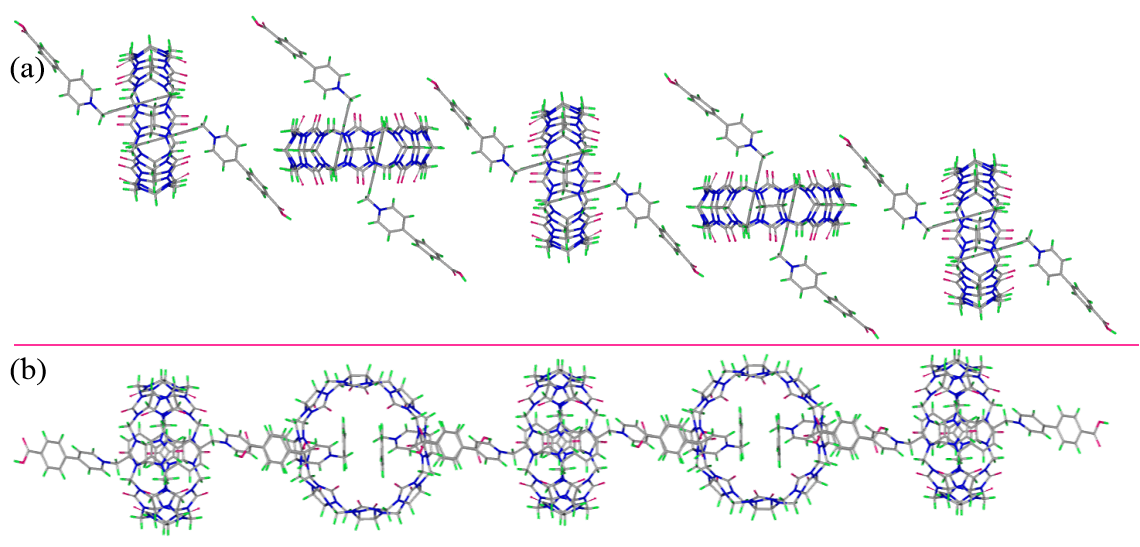


**Figure S5.** Crystal structure of one-dimensional supramolecular chain constructed of the **NapA** and Q[8].


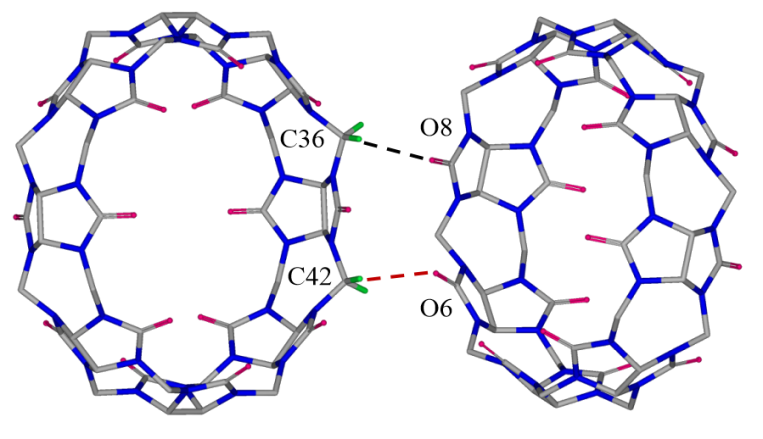


**Figure S6.** The hydrogen bonds between the carbonyl oxygens at the portals of the Q[8] host and the hydrogen atom on the methylene of the adjacent Q[8] (**Nap1_2_**@Q[8]).


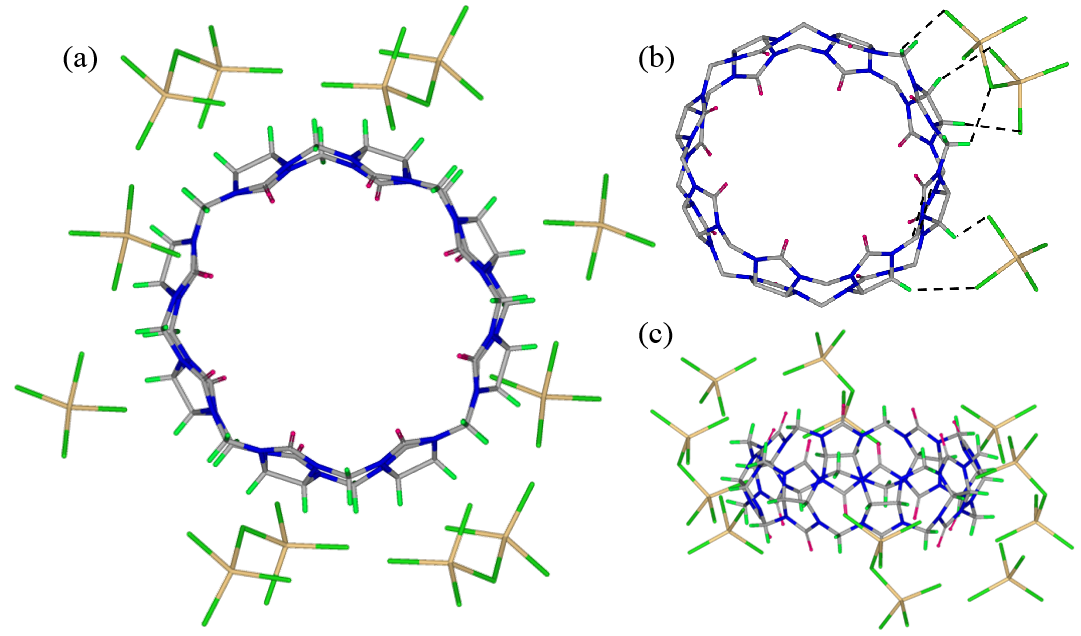


**Figure S7.** Detailed interactions between Q[8] and [CdCl_4_]^2−^ anions (**Nap1_2_**@Q[8]).

**Figure S8.** ^1^H NMR spectra of **NapA** in DMSO at 25 °C.

**Figure S9.** ^13^C NMR spectra of **NapA** in D_2_O at 25 °C.

**Figure S10.** ^1^H-^1^H COSY spectra of **NapA** in D_2_O at 25 °C


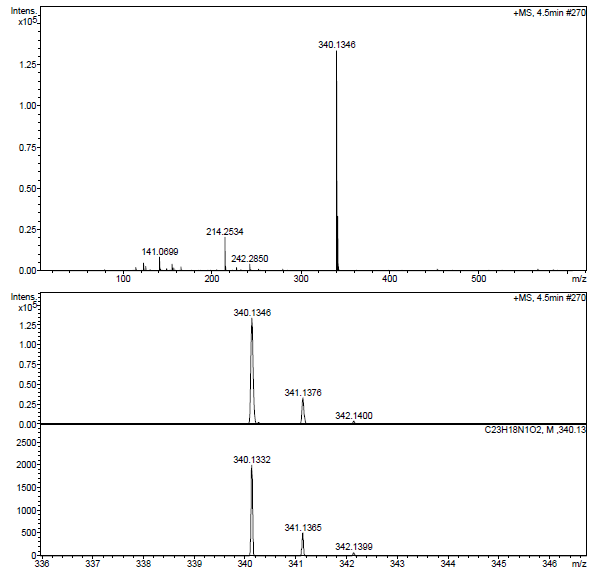


**Figure S11.** HR-MS (ESI): Calcd for **NapA** C_23_H_18_NO_2_^+^: 340.1332 [M-Br]^+^. Found: 340.1346.


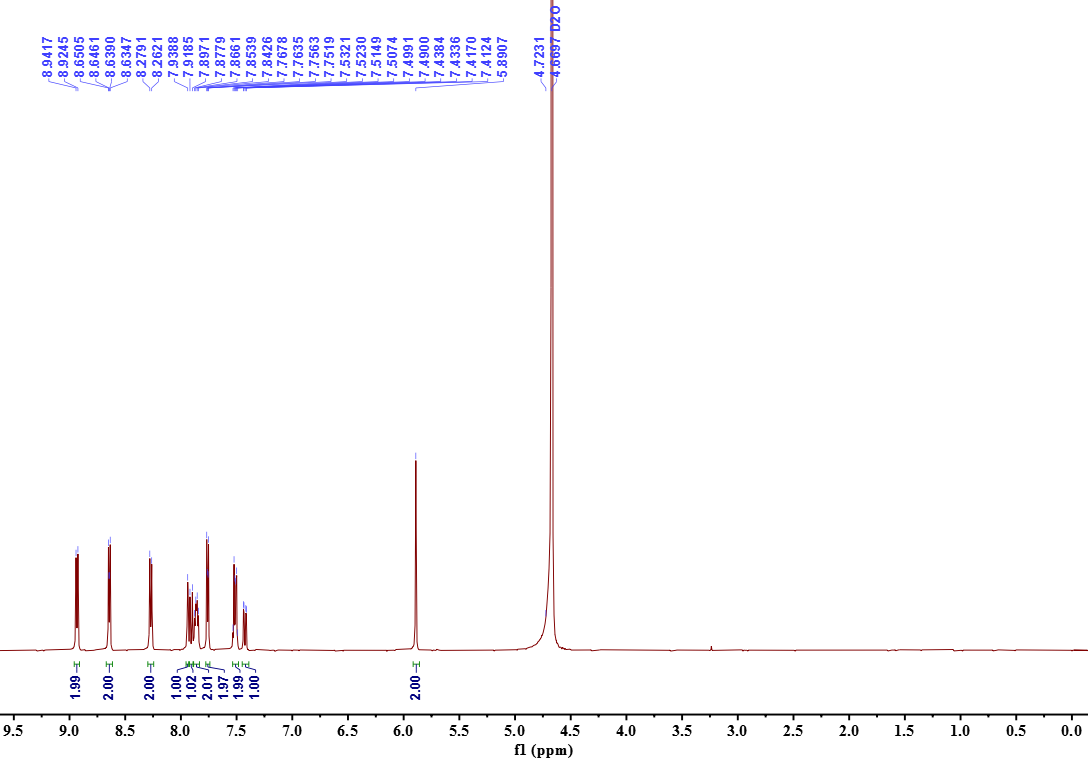


**Figure S12.** ^1^H NMR spectra of **Nap1** in DMSO at 25 °C.


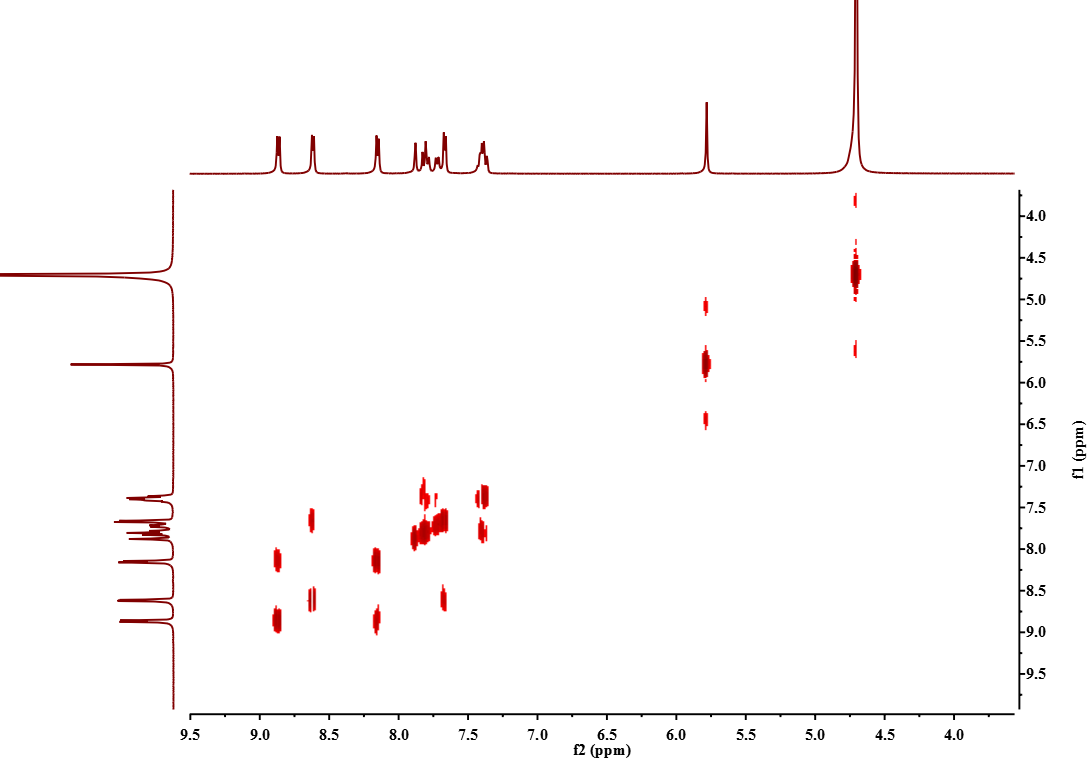


**Figure S13.** ^1^H-^1^H COSY spectra of **Nap1** in D_2_O at 25 °C.


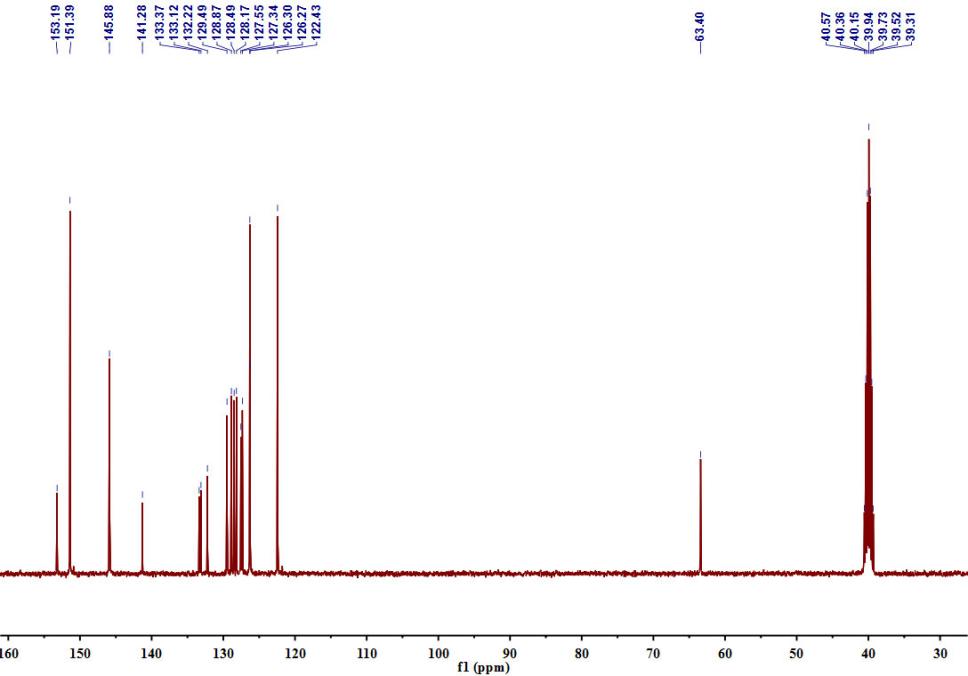


**Figure S14.** ^13^C NMR spectra of **Nap1** in D_2_O at 25 °C.


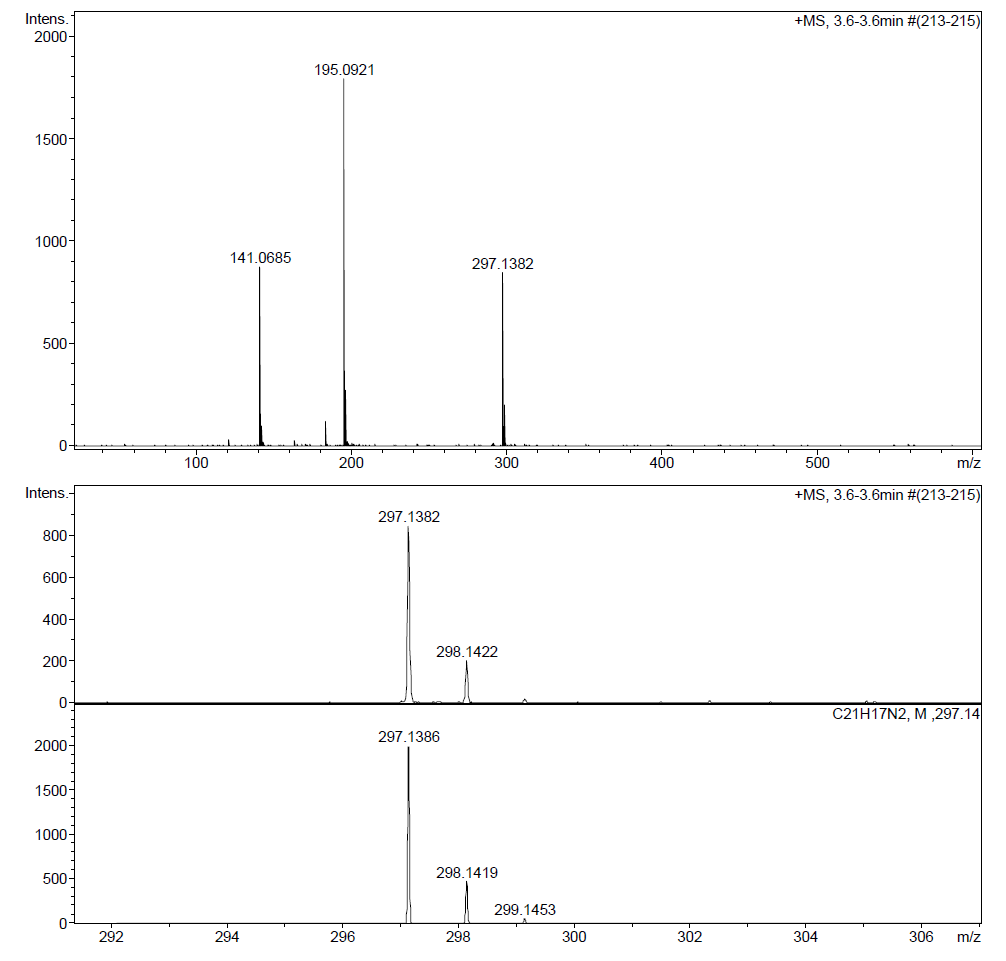


**Figure S15.** HR-MS (ESI): Calcd for **Nap1** C_21_H_17_N_2_: 297.1386 [M-Br]^+^. Found: 297.1382.

**Table S1.** ITC measurements of the thermodynamics of **NapA**_2_@Q[8] and **Nap1**_2_@Q[8] interactions in aqueous solution at 298.15 K.

| Experiment | NapA_2_@Q[8] | Nap1_2_@Q[8] |
| --- | --- | --- |
| Binding constant (*K_a_*) (10^10^ M^−2^) | 2.14 ± 0.62 | 1.48 ± 0.45 |
| Enthalpy change (Δ*H*) (kJ/mol) | -36.17 ± 2.12 | -22.71 ± 1.37 |
| Entropy change (-*T*Δ*S*) (kJ/mol) | 13.71 | 9.53 |
| Gibbs free energy (Δ*G*) (kJ/mol) | 22.46 | 13.18 |
